# Changes in potential pathogenicity-associated proteins of *Helicobacter cinaedi* upon infection of macrophage cells

**DOI:** 10.3389/fmicb.2025.1640829

**Published:** 2025-08-25

**Authors:** A. K. Voronina, B. A. Efimov, M. V. Malakhova, P. V. Shnaider, O. M. Ivanova, M. Bogomiakova, V. O. Shender, M. A. Lagarkova, G. P. Arapidi

**Affiliations:** ^1^Lopukhin Federal Research and Clinical Center of Physical-Chemical Medicine of Federal Medical Biological Agency, Moscow, Russia; ^2^Federal State Autonomous Educational Institution of Higher Education “N.I. Pirogov Russian National Research Medical University” of the Ministry of Health of the Russian Federation, Moscow, Russia; ^3^Shemyakin-Ovchinnikov Institute of Bioorganic Chemistry of the Russian Academy of Sciences, Moscow, Russia; ^4^Moscow Institute of Physics and Technology (National Research University), Dolgoprudny, Russia

**Keywords:** *Helicobacter cinaedi*, macrophages, proteome analysis, virulence factor, mass-spectrometry, bacterial infection of eukaryotic cells, pathogen escape from cellular immunity, bacterial pathogenicity

## Abstract

**Introduction:**

*Helicobacter cinaedi* is a spiral-shaped Gram-negative, enterohepatic bacterium classified as a conditional pathogen (pathogenicity group 2). It is known to cause bacteremia and a variety of other diseases in humans. In particular, *Helicobacter cinaedi* has been shown to impair intracellular cholesterol metabolism when interacting with macrophages, leading to foam cell formation. M1-macrophages transformed into the foam cell phenotype contribute to atherosclerotic plaques, suggesting a potential link between *H. cinaedi* infection and atherosclerosis development.

**Methods:**

To uncover protein factors involved in *H. cinaedi* pathogenesis, we performed a detailed mass spectrometric analysis of the proteome of strain BAA-847. This study represents the first comprehensive analysis of the bacterium’s proteome under standard culture conditions and after infection of M1-type macrophage cells.

**Results:**

We identified 1,575 proteins in the *H. cinaedi* proteome, 109 of which were differentially upregulated after macrophage infection. Functional analysis revealed roles for these proteins in immune evasion, intracellular survival, and potential pathogenicity. Beyond known virulence factors (HcaA, Cdt, AhpC), we identified poorly characterized proteins with toxic or immunomodulatory functions. Notably, some upregulated proteins enable cholesterol utilization as a carbon source, while others may participate in a toxin injection mechanism disrupting host cell metabolism—potentially linked to foam cell formation.

**Conclusion:**

Our findings provide new insights into *H. cinaedi* pathogenicity, highlighting previously unexplored virulence mechanisms. The identified proteins could serve as targets for further research into *H. cinaedi*-associated diseases, including atherosclerosis.

## Introduction

Cardiovascular diseases rank first in mortality according to the World Health Organization.^1^ Among heart and vascular diseases, atherosclerosis is the largest contributor to mortality (Björkegren and Lusis, 2022). According to the “response to injury” hypothesis, atherosclerosis may be induced by impaired lipoprotein metabolism, in which macrophages fail to clear modified low-density lipoproteins (LDL), leading to their transformation into foam cells, which eventually die (Hou et al., 2023). Several published studies suggest that disturbances in LDL metabolism in macrophages can be caused by the intracellular pathogen, *Helicobacter cinaedi*, but it’s definitive role in the disease development remains unclear. It is hypothesized that after oral infection, *H. cinaedi* may translocate into vascular tissue, triggering proatherosclerotic inflammatory processes. Specifically, foam cell formation could be linked to the bacterium’s ability to reduce the level of ABCG1, a protein involved in cholesterol efflux from macrophages (Khan et al., 2012, 2014).

Although several research articles (Khan et al., 2012, 2014) and even reviews (Voronina and Arapidi, 2024; Shimizu and Shimizu, 2016) have been devoted to the study of this bacterium, only four *H. cinaedi* virulence factors have been described in the literature: cytolethal distending toxin (Cdt) (Taylor et al., 2003), alkyl hydroperoxide reductase (AhpC) (Charoenlap et al., 2012), *Helicobacter cinaedi* autotransporter protein (HcaA) (Aoki et al., 2023) and Cinaedi atherosclerosis inflammatory protein (CAIP) (Paolini et al., 2024). Cdt is a lethal toxin that induces apoptosis and cell cycle arrest in host cells. AhpC is an enzyme involved in oxidative stress defense, promoting the bacterium’s survival within the host cell. HcaA is an autotransporter protein, a type V secretory system secretory protein (T5SS), which facilitates adhesion to host cells. Additionally, *H. cinaedi* is also known to possess a type VI secretion system (Goto et al., 2012). CAIP is a *H. cinaedi* protein that is hypothesized to promote LDL accumulation in macrophage.

Investigation of specific virulence factors is an important task in the study of pathogenic microorganisms. Comparative proteomic analysis is a valuable tool for obtaining such data, as it allows for the comparison of protein expression profiles in bacteria under culture conditions versus after host cell infection. For example, Lamberti’s group employed proteomic methods to examine how the protein profile of the *Bordetella pertussis* changes following infection of macrophage cells (Lamberti et al., 2016). The study revealed an enrichment of chaperone proteins GroEL, GroES, and ClpB, the RNA-binding protein Hfq, and the BP206 protein, which is involved in DNA repair after infection. In another study, the ability of *Salmonella enterica* to affect the endosomal system of macrophage cells via its T3SS was investigated using comparative proteomic analysis (Noster et al., 2019). After infection, increased amounts of proteins associated with nutritional starvation were observed in the proteome of the T3SS-defective strain compared to the wild-type strain. Mass spectrometry and two-dimensional gel electrophoresis are commonly used to analyze the protein profiles. For instance, in a study by Professor Govorun’s group, they examined the phase transition of *Mycoplasma gallisepticum* during invasion of eukaryotic cells (Matyushkina et al., 2016). After infection, a significant reorganization of the bacterial proteome was observed, along with an enhancement of functions related to hydrogen peroxide production, glycerol utilization, and SpxA protein regulation.

A major challenge in such investigations is obtaining intracellular bacteria of high purity, free from contamination by extracellular bacteria that could distort the proteomic profile (Li et al., 2010). In the studies mentioned above, the authors focused on eliminating extracellular bacteria after macrophage infection and validating the purity of the intracellular bacteria. To achieve this, they used antibiotics that do not penetrate mammalian cells, such as gentamicin (Matyushkina et al., 2016; Noster et al., 2019) or polymyxin B sulfate (Lamberti et al., 2016). Several methods can be employed to validate the purity of intracellular bacteria, including seeding the supernatant remaining after washing the cells to remove extracellular bacteria, or visualizing the bacteria during infection using immunofluorescence staining (Lamberti et al., 2016). In the present study, we conducted a comparative proteomic analysis of *H. cinaedi* in a macrophage infection model to identify protein factors potentially contributing to bacterial pathogenesis.

## Materials and methods

### Reagents and solutions

A 0.01 M Phosphate-Buffered Saline (PBS) solution was prepared by diluting PBS tablets (Thermo Fisher, USA) in milli-Q distilled water. A 0.05% trypsin solution was prepared by diluting a 0.25% trypsin–EDTA solution (Thermo Fisher, USA) in RPMI advanced medium (PanEco, Russia).

### *Helicobacter cinaedi* strain and growth conditions

*Helicobacter cinaedi* BAA-847 (purchased from the American Type Culture Collection, ATCC-BAA-847) was used for all experiments described. *H. cinaedi* was grown on Columbia Broth Base (Himedia, India) at 37°C. To create microaerophilic conditions, 75% of the volume in the anaerobic jar (Schuett, Germany) was replaced with a gas mixture composed of N_2_ (84.18%), H_2_ (10.51%), and CO_2_ (5.31%).

### Determination of *Helicobacter cinaedi* growth dynamics

To determine the growth dynamics, the bacterium was cultured for 1 week on Columbia broth medium (Himedia, India). Every day the optical density of the culture medium with the bacterium was measured using an AMR-100A spectrophotometer (Allsheng, China) at a wavelength of 600 nm (OD_600_). Pure culture medium served as the control solution for calibration. Each day, the number of viable cells was determined by seeding duplicate serial 10-fold dilutions (10^−1^ to 10^−8^) of the culture in the Columbia broth followed by plating on Columbia agar (bioMérieux, France) supplemented with 5% sheep blood. After incubation a for 4–7 days in microaerophilic conditions, colonies were counted (Supplementary Figures 1C–E), and CFU/mL was calculated as:

$$\mathrm{CFU}/\mathrm{mL}=\frac{Volumeplated\;\left(mL\right)}{Numberofcolonies\times Dilutionfactor}$$

### Determination of gentamicin minimum inhibitory concentration

To determine the minimum inhibitory concentration (MIC) of gentamicin, 1 mL of bacterial culture in the exponential growth phase was added to each well of a 6-well culture plate (SPL Lifesciences, South Korea). Gentamicin was then added to the wells at the following final concentrations: 31.25 μg/mL, 62.5 μg/mL, 125 μg/mL, 250 μg/mL, and 500 μg/mL. The plate was incubated under microaerophilic conditions for 2.5 h. Following incubation, a microbiological loop with a volume of 10 μL was used to transfer a sample from each well into a separate vial containing 40 mL of fresh liquid nutrient medium. These cultures were incubated for an additional 7 days. The MIC was determined visually as the lowest concentration of gentamicin that completely inhibited bacterial growth.

### Macrophages obtained from donor’s peripheral blood mononuclear cells

This study was reviewed and approved by the Ethics Committee of the Lopukhin Federal Research and Clinical Center of Physical–Chemical Medicine of the Federal Medical Biological Agency. The healthy donor provided written informed consent to participate in this study.

BD Vacutainer CPT Mononuclear Cell Preparation Tube—Sodium Heparin (BD Bioscience, US) was used to obtain peripheral blood mononuclear cells (PBMC). For PBMC isolation, blood stored at +4°C for no more than 8 h from the time of collection from the donor was used. Vacutainers were centrifuged at 800 g for 20 min in A-4-44 swinging-bucket rotor (Eppendorf, Germany). The interphase ring containing monocytes was collected from the stratified fluid and transferred to a 15-mL Falcon centrifuge tube. The cells were then washed three times with 8 mL of 0.01 M PBS, followed by centrifugation at 300*g* for 15 min at room temperature after each wash.

Serum for PBMC culture was obtained from blood of the same donor as follows: blood was collected in a Vacuette tube Serum Clot Activator (Greiner-Bio-One, Germany) was incubated at room temperature for 1 h, the clot was precipitated by centrifugation at 800*g* for 20 min in A-4-44 swinging-bucket rotor (Eppendorf, Germany), then the serum was withdrawn into a sterile tube and inactivated in a water bath for 1 h at 56°C. PBMC were cultured for 7 days in a CO₂ incubator at 37°C in Advanced RPMI medium (PanEco, Russia) supplemented with 10% donor serum, 10 μg/mL gentamicin, and cytokines: GM-CSF (12 ng/mL) and IFN-*γ* (100 ng/mL) to induce differentiation of monocytes into M1 macrophages.

### Verification of macrophage differentiation to M1 phenotype

The differentiation of macrophages into the M1 phenotype was evaluated using an Acea Novocyte 3,000 flow cytometer (Novocyte, Agilent, USA). To confirm the phenotype, antibodies targeting the surface markers CD14, CD80, and CD86 were used, as high expression of these markers is characteristic of M1 macrophages (Oates et al., 2022). Macrophage cells were detached from the culture plate by incubating with 0.05% trypsin solution for 10 min. Following detachment, the cells were washed once in PBS and centrifuged at 300*g* for 2 min. The cells were then divided into three groups: one control group and two experimental groups.

For antibody staining, Alexa Fluor 647 anti-human CD14 (Sony Biotechnology, US, Cat. № 2435635) and PE anti-human CD80 (Sony Biotechnology, US, Cat. № 2126040) antibodies were added to one experimental group, and BD Horizon BV605 Mouse Anti-Human CD86 (BD Biosciences, US, Cat. № 562999) antibody was added to the second experimental group. Antibodies were used at a dilution 1:200 and incubated in the dark for 15 min. After incubation, unbound antibodies were removed by centrifugation at 300*g* for 2 min, and the cells were resuspended in 110 μL of 0.01 M PBS.

Flow cytometry analysis was performed using the following settings: For Alexa Fluor 647, excitation was at 640 nm, with emission captured through a 675/30 nm filter; for PE, excitation was at 488 nm, with emission captured through a 572/28 nm filter; and for BV605, excitation was at 488 nm, with emission captured through a 615/20 nm filter.

### Infection of macrophage cells with the *Helicobacter cinaedi* bacterium

M1-type macrophages were cultured in eight-well slide vials (SPL Lifesciences, South Korea) for staining and analysis under a fluorescence microscope, or in 3.5-cm diameter Petri dishes (SPL Lifesciences, South Korea) to obtain the proteome. A bacterial cell suspension was added to the one-week macrophage culture. The initial seeding density of macrophage cells was at 250,000 cells per well in the vials or 2 million cells per Petri dish.

Before infection with bacteria, the macrophage cell culture was washed five times with sterile PBS, and the medium was replaced with RPMI advanced medium supplemented with 10% donor serum, excluding gentamicin. Only a portion of the required medium volume was added, specifically 375 μL per well in the slide vials or 500 μL per Petri dish.

On the 3rd day of bacterial cultivation (beginning of the exponential growth phase OD_600_ = 0.5), the bacterial culture was used to infect the macrophages. The bacterial culture was collected sterilely into a centrifuge tube, and the bacterial cells were pelleted by centrifugation at 5,000*g* for 5 min. The supernatant was discarded, and the bacterial pellet was resuspended in half the volume of RPMI advanced medium supplemented with 10% donor serum. A total of 125 μL of the resulting bacterial suspension was added to the macrophages in each slide vial, or 1 mL was added to each Petri dish, resulting in a multiplicity of infection (MOI) of approximately 5 bacteria per macrophage cell. After infection, the slide vials and Petri dishes were incubated in a СО_2_ incubator at 37°C for 24 h.

For staining and fluorescence microscope analysis, macrophage cells were used as a negative control, to which RPMI advanced medium with 10% FBS (without gentamicin) was added instead of bacterial suspension.

**Figure 1:**
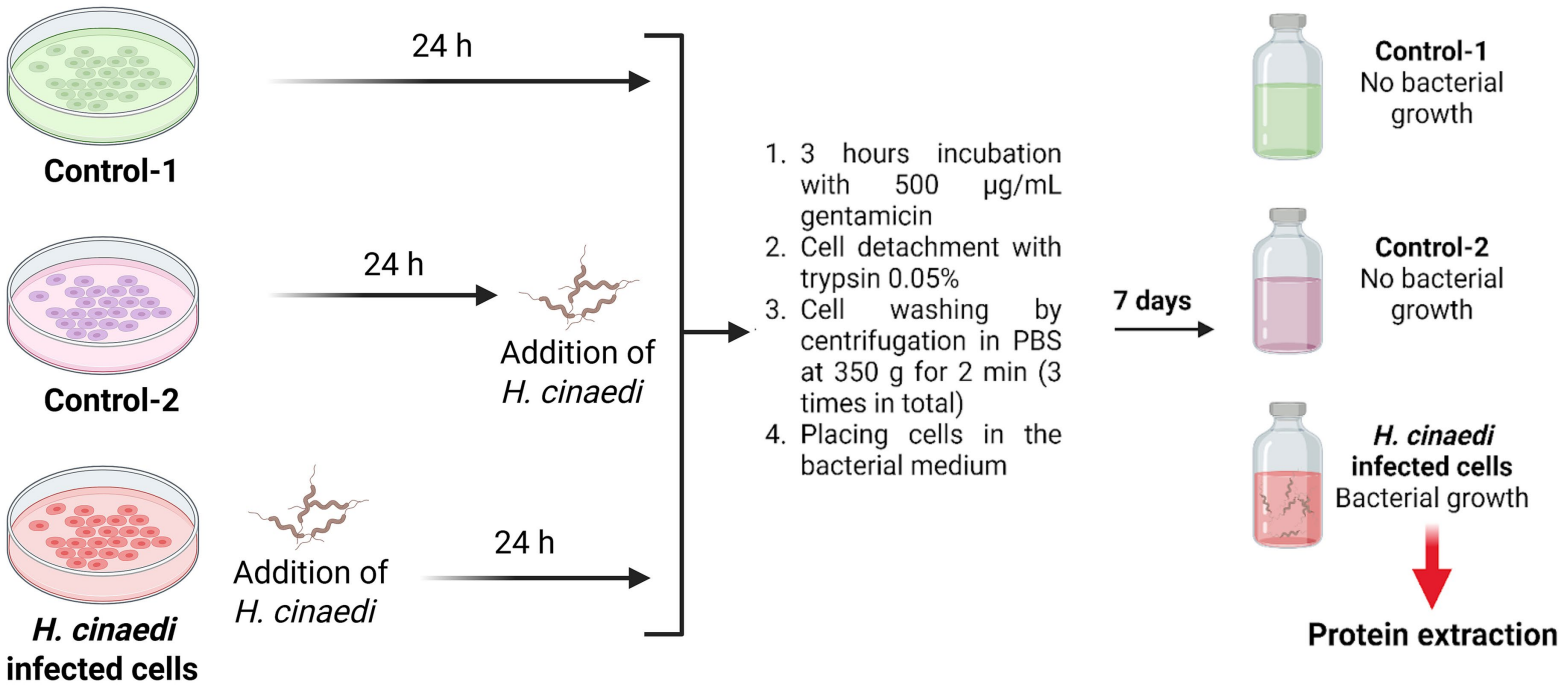
Scheme of the experiment for macrophage cell infection with *Helicobacter cinaedi* for proteome isolation. After incubation with gentamicin, detachment with trypsin and washing with PBS, the macrophage cells were placed in bacterial medium where they were subsequently lysed and the surviving intracellular bacteria were allowed to proliferate. Created with BioRender.com.

To obtain the bacterial proteome after infection of macrophage cells, three independent biological repeats were performed, each at least 1 week apart. In each experiment repeat, there were three samples: control-1, control-2 (antibiotic efficacy control), and the experimental sample (Figure 1). First, the experimental sample was infected with a three-day-old culture of *H. cinaedi,* and all three samples were maintained at 37°C in a 5% CO_2_ atmosphere for 24 h. The control samples were left uninfected. Cells from the same three-day-old culture of *H. cinaedi* were used to obtain the bacterial proteome under culture conditions. Next, 500 μg/mL gentamicin (double the MIC) was added to all three samples. *H. cinaedi* was added to the antibiotic efficacy control (control-2) immediately afterward. All three samples were then incubated with gentamicin for 3 h in a СО_2_ incubator at 37°C. Macrophage cells were then detached using 0.05% trypsin solution, washed three times by centrifugation in PBS at 350*g* for 2 min, and transferred to bacterial medium. The samples were cultured in an anaerobic jar for 1 week. At the end of this period, bacterial growth was observed in the experimental sample with no bacterial growth in either control sample. The bacterial proteome was then isolated.

### Macrophage cells staining with DAPI and oil red-O (ORO), followed by fluorescence microscope analysis

A 1:50,000 DAPI solution was prepared by diluting DAPI (Sigma-Aldrich, US) in Milli-Q distilled water. A 0.3% Oil Red-O (ORO) solution was prepared by dissolving Oil Red-O powder (Sigma-Aldrich, US) in isopropanol, followed by the addition of milli-Q distilled water to achieve an isopropanol:water ratio of 3:2. The resulting solution was passed through filter paper to remove any ORO dye particles.

Culture medium was removed from the cells in the slide vials, and each cell was washed once with 200 μL of 0.01 M PBS. Then, 200 μL of 4% Paraformaldehyde (PFA) solution (Sigma-Aldrich, US) was added to each cell and incubated for 30 min to fix them. After fixation, the cells were washed twice with 200 μL of PBS and once with 200 μL of 60% isopropanol. Next, 70 μL of the 0.3% ORO solution prepared in a 3:2 isopropanol:water mixture was added to each well, and the cells were incubated for 8 min in the dark.

After ORO staining, the cells were washed three times with 200 μL of 60% isopropanol followed by two washes with 200 μL of 0.01 M PBS. Subsequently, 200 μL of DAPI solution was added to each slide vial, and the cells were incubated in the dark for 8 min. After staining, cells were washed three times with 200 μL of 0.01 M PBS. The slide walls were then removed, and excess liquid was carefully blotted using filter paper. To mount the samples, 10 μL of glycerol was placed at the center of each slide, and coverslips were applied. Images were captured using a Nikon Eclipse Ni-E fluorescence microscope (Nikon Instruments Inc., USA) using DAPI, FITC, and TRITC channels. After staining, cells were washed three times with 200 μL of 0.01 M PBS. The slide walls were then removed, and excess liquid was carefully blotted using filter paper. To mount the samples, 10 μL of glycerol was placed at the center of each slide, and coverslips were applied. Images were captured using a Nikon Eclipse Ni-E fluorescence microscope (Nikon Instruments Inc., USA) using DAPI, FITC, and TRITC channels.

### Protein isolation, trypsinolysis and preparation for MS analysis

Bacterial cells were washed three times from the medium in 0.01 M PBS: centrifuged for 10 min at 5,000*g* at +4°C. Bacterial cells were lysed in 500 μL of 10% sodium dodecyl sulfate solution (SDS, Sigma-Aldrich, US) in PBS supplemented with 5 μL protease inhibitor cocktail (Promega, US), and 2 μL Nuclease mix (Cytiva, US). This mixture was incubated for 1 h on ice, after which Sample Buffer [2.5 mM EDTA (Sigma-Aldrich, US), 0.1 M Tris (Sigma-Aldrich, US) and 8 M urea (Cytiva, US)] was added at a ratio of 2 parts buffer to 1 part mixture. After a 30-min incubation at room temperature, cell debris was removed by centrifugation at 15,000*g* for 10 min at 4°C. Protein concentration in the resulting supernatant was determined using the Quick Start™ Bradford Protein Assay Kit 1 (Bio-Rad, US) according to the manufacturer’s instructions.

To reduce disulfide bonds, the lysate was incubated with 5 mM dithiothreitol (DTT; Sigma-Aldrich, US) for 30 min at room temperature on a TS-100C thermoshaker (Biosan). Subsequently, cysteine thiol groups were alkylated by adding 1 M iodoacetamide to a final concentration of 10 mM, followed by incubation for 20 min under the same conditions. To minimize the interference of residual urea with trypsin digestion, the solution was diluted 4-fold with 50 mM ammonium bicarbonate (Sigma-Aldrich, US). Trypsin (Promega, US) was added at a ratio of 1 μg of enzyme per 100 μg of total protein, and the mixture was incubated for 12 h at 37°C on a thermoshaker. Proteolysis was terminated by the addition of 2 μL of 100% trifluoroacetic acid (TFA), adjusting the pH to < 2, followed by a 1-h incubation at 37°C.

For desalting of the resulting tryptic hydrolysate, sample columns were prepared according to the method described by Rappsilber et al. (2007). Following sample application, the columns were centrifuged at 300*g* for 10 min. The samples were then desalted with 100 μL of 0.2% trifluoroacetic acid (TFA), followed by elution using 50 μL of a solution containing 18% ammonium hydroxide (NH₄OH) and 50% acetonitrile (ACN), under the same centrifugation conditions. The eluates were subsequently dried using a Concentrator Plus centrifugal evaporator (Eppendorf, Germany).

### Chromatography-mass spectrometric analysis and protein identification

Mass spectra were acquired using a Q Exactive HF-X Hybrid Quadrupole-Orbitrap Mass Spectrometer (Thermo Fisher Scientific, USA) following previously described protocols (Rappsilber et al., 2007; Shender et al., 2024). Peptide samples were loaded onto 50-cm in-house packed analytical columns containing C18, 3 μm Acclaim PepMap 100 resin (Thermo Fisher Scientific), using an Ultimate 3,000 Nano LC System (Thermo Fisher Scientific) coupled online to the mass spectrometer.

Data acquisition was performed in a data-dependent acquisition (DDA) mode with automatic switching between full MS (MS1) scans and up to 15 MS/MS scans (TopN method). The MS1 scan range was set from 300 to 1,200 *m/z*, with a resolution of 60,000 at *m/z* 200, a maximum injection time of 60 ms, and an AGC (automatic gain control) target of 3 × 10^6^. Precursor ions were isolated using a 1.4 *m/z* window, with the first mass fixed at 100.0 *m/z*. Fragmentation was performed using higher-energy collisional dissociation (HCD) with a normalized collision energy of 28 eV. MS/MS scans were acquired with a resolution of 15,000 at *m/z* 400, a scan range of 200–2,000 *m/z*, a maximum ion injection time of 30 ms, and an AGC target of 1 × 10^5^.

Raw LC–MS/MS data from Q Exactive HF-X mass spectrometer were converted to mgf peaklists with MSConvert from the ProteoWizard Software Foundation. The following parameters were used: “--mgf --filter peakPicking true [1,2].” To thoroughly identify peptides and proteins, we applied an approach based on the use of two search algorithms: MASCOT (version 2.5.1) and X! Tandem (ALANINE, 2017.02.01) against the combined UniProtKB protein sequence database, *Helicobacter cinaedi* and *Bos Taurus* taxon (culture medium included acidic casein peptone and bovine extract) downloaded from the UniProt knowledgebase web resource (UniProtKB^2^). The precursor and fragment mass tolerance were set at 20 ppm and 0.04 Da, respectively. Database search parameters included the following: tryptic digestion with 1 possible missed cleavage; static modification for carbamidomethyl (C); dynamic/flexible modifications for oxidation (M). For X! Tandem, additional parameters were selected to check for protein N-terminal residue acetylation, peptide N-terminal glutamine ammonia loss, or peptide N-terminal glutamic acid water loss. Result files from the search engines were then submitted to Scaffold 5 software (version 5.1.0) for validation and meta-analysis. We used the local false discovery rate scoring algorithm with standard experiment-wide protein grouping. For the evaluation of peptide and protein hits, a false discovery rate of 5% was selected for both. False positive identifications were based on reverse database analysis.

A total of 109 differentially represented proteins were selected for detailed analysis based on the following filtration criteria: (1) proteins exhibited a ≥20% increase in abundance in bacterial cells following infection; (2) the total number of peptide-spectrum matches (PSMs) across all samples was ≥5, ensuring sufficient protein representation; (3) proteins were identified in at least two out of three biological replicates post-infection, indicating reproducibility; and (4) the difference in protein abundance between infected and culture-grown bacterial cells yielded a *p*-value < 0.05 by the Mann–Whitney test. These criteria were established to ensure the inclusion of only reliably detectable proteins that showed statistically significant changes in abundance following macrophage infection.

## Results and discussion

### Infection of macrophage cells with *Helicobacter cinaedi*

**Figure 2:**
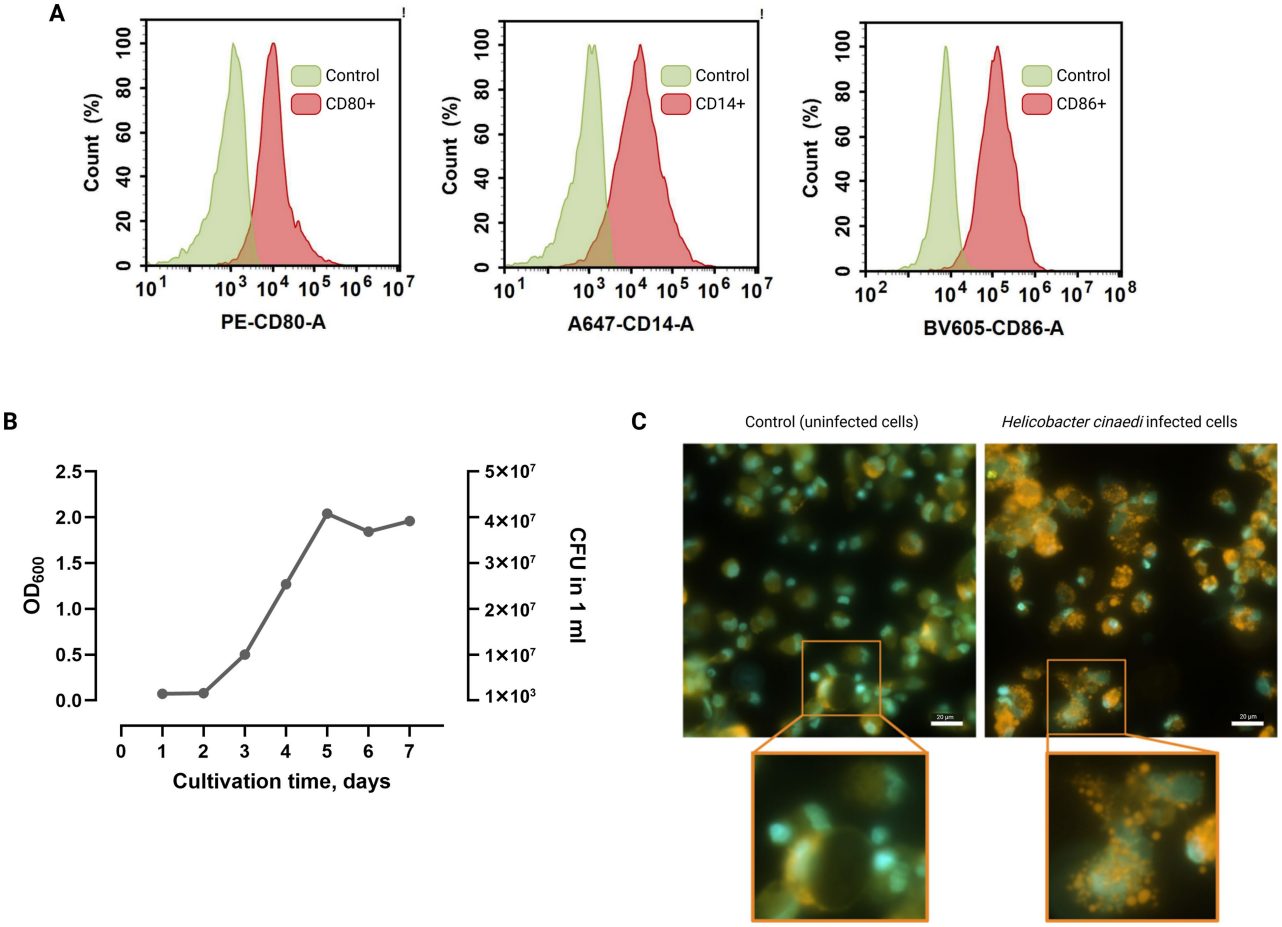
**(A)** Flow cytometric analysis of macrophage cells stained with antibodies against the macrophage membrane markers CD14, CD80, and CD86. Simultaneous expression of these markers confirms the acquisition of the M1 phenotype by the macrophages. Average of two repeats. **(B)** Growth dynamics of *H. cinaedi* over 1 week. **(C)** Microscopic images of uninfected and *H. cinaedi*-infected macrophages, stained with DAPI (blue) and Oil Red-O (orange). Infected macrophages display brightly stained lipid granules in the cytoplasm, indicative of a foam cell phenotype. Insets show magnified views of the boxed areas. Scale bar, 20 μm.

To determine growth dynamics, *H. cinaedi* was cultured for 1 week (Figure 2B). Under the applied culture conditions, the exponential growth phase began on day two, with the mid-log phase occurring on day three, consistent with previously reported data (Schmitz et al., 2016). On the third day, the optical density at 600 nm (OD₆₀₀) reached 0.5, corresponding to approximately 1 × 10^7^ CFU/mL. Based on these observations, bacterial suspensions harvested on the third day of growth were used for macrophage infection experiments.

To eliminate extracellular *H. cinaedi* following infection, the minimum inhibitory concentration (MIC) of gentamicin was determined to be 250 μg/mL (Supplementary Figures 1A,B). For infection experiments, a concentration of 500 μg/mL (2 × MIC) was used to ensure effective elimination of extracellular bacteria.

To generate M1 phenotype macrophages, monocytes were isolated from the peripheral blood of a healthy donor and differentiated under specific polarization conditions. Flow cytometric analysis confirmed successful differentiation, with the majority of cells exhibiting surface markers characteristic of the M1 macrophage phenotype (Figure 2A).

Prior to infection, macrophage cells were washed five times with sterile PBS, and the culture medium was replaced with RPMI Advanced medium supplemented with 10% gentamicin-free donor serum. Bacterial cultures harvested on the third day of growth were used for infection at a multiplicity of infection (MOI) of approximately 5 bacteria per macrophage. Fluorescence microscopy of infected cells stained with DAPI and Oil Red O (ORO) revealed a foam cell phenotype, indicating successful infection by *H. cinaedi* (Figure 2C).

### Protein profile changes with decrease in AhpC and CDT content in the *Helicobacter cinaedi* proteome after infection of macrophage cells

*Helicobacter cinaedi* proteins were isolated in four independent experiments, each conducted at least 1 week apart. In three of these experiments, proteins were isolated both from bacteria cultured under standard conditions and from bacteria recovered after infection of macrophage cells, growing for a week in normal culture conditions prior to protein extraction for proteomics. In the fourth experiment, proteins were isolated only from bacteria grown under culture conditions. Each infection experiment included two controls, one of which served as an antibiotic efficacy control (Figure 1, Control-2). A total of 1,575 *H. cinaedi* proteins were identified (Supplementary Table 1). Of the total proteins, 1,030 (65%) were consistently identified across all seven samples—four from cultured bacteria and three from infected macrophage cells. For reference, the genome of the *H. cinaedi* strain BAA-847 encodes 2,322 protein-coding genes (Miyoshi-Akiyama et al., 2012).

**Figure 3:**
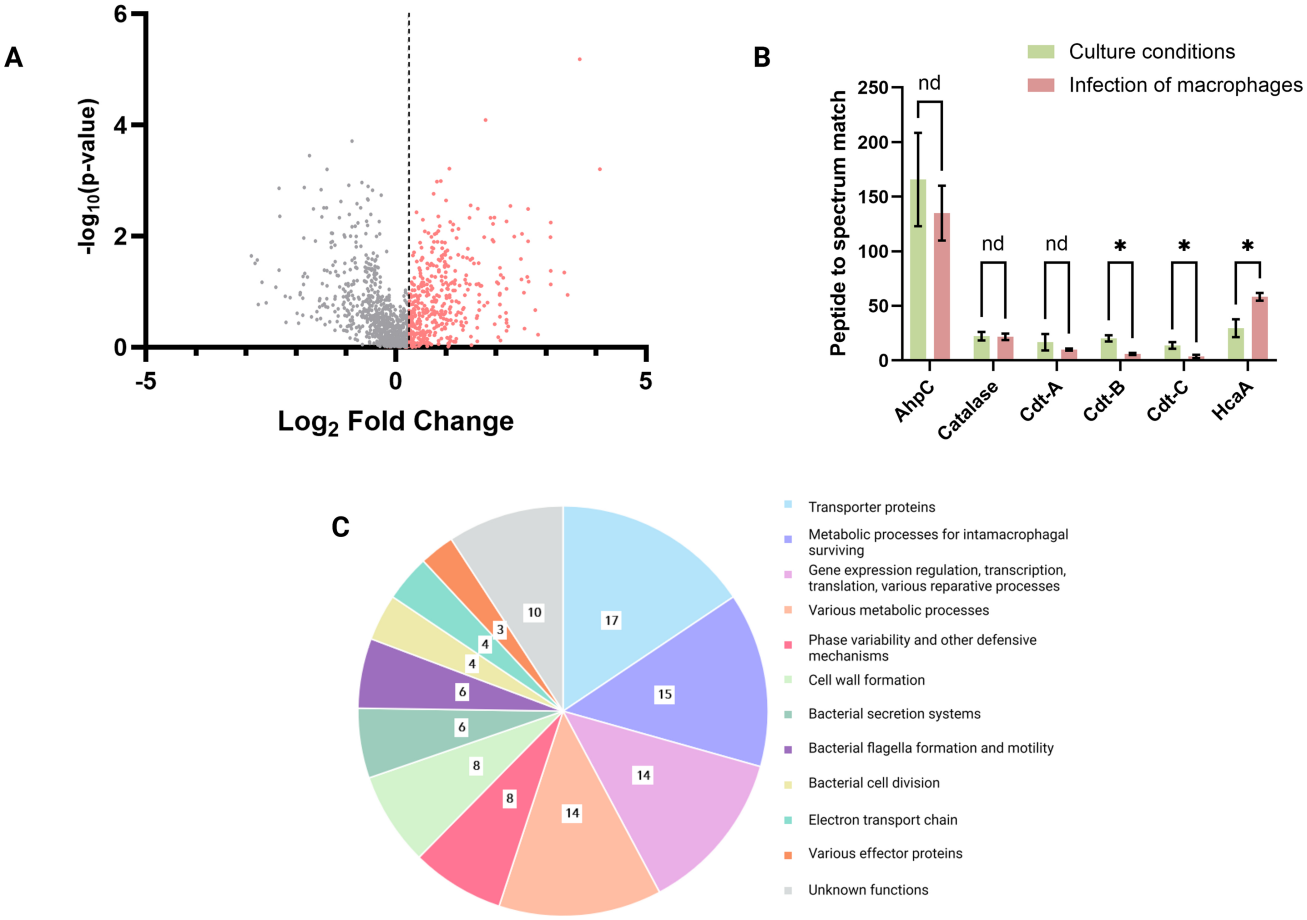
**(A)** Representation of proteins identified in the *H. cinaedi* proteome under culture conditions and after infection with macrophages, among which 1,234 proteins that were overrepresented by 20% or more in *H. cinaedi* cells after infection with macrophages compared to cells grown under culture conditions; threshold: Log_2_ Fold change = 0.263. **(B)** Changes in the representation of known pathogenicity factors of *H. cinaedi* in the proteome after infection. In addition to well-characterized pathogenicity factors such as alkyl hydroperoxide reductase (AhpC), the three subunits of cytolethal distending toxin (Cdt), and *Helicobacter cinaedi* autotransporter protein (HcaA), catalase is represented in the diagram. Catalase is present at rather high levels (in the first third among all identified peptides by Peptide to spectrum match) in both the culture-grown and infected bacterial proteomes, and its activity may compensate for the loss of AhpC function. It is also possible that in addition to catalase, some other bacterial factors are involved in the defense against oxidative stress, which are not yet known. *p < 0.05; nd, not defined (Mann–Whitney test). **(C)** Functional distribution of the 109 most represented bacterial proteins in the *H. cinaedi* proteome after infection of macrophage cells.

Among the identified proteins, 1,234 were found to be overrepresented by 20% or more in *H. cinaedi* cells following macrophage infection compared to those grown under culture conditions (Figure 3A). From this subset, we selected 109 proteins based on the following criteria: (i) a minimum total of five peptide-to-spectrum matches (PSMs) across all samples, (ii) consistent detection in at least two out of three post-infection proteome replicates, and (iii) a statistically significant difference in representation between post-infection and cultured conditions (*p* < 0.05, Mann–Whitney test). The list of these 109 proteins, along with brief functional annotations, is provided in Supplementary Table 2.

In an article on the *H. cinaedi* CAIP pathogenicity factor, the authors reported that this protein is a product of the bacterium’s *napA* gene (D’Elios et al., 2017). In our proteome we found only two *napA* gene products—Neutrophil-activating protein (I7H4K9) and Ni/Fe hydrogenase alpha subunit (I7GT85) and thus, it seems that the pathogenicity factor CAIP is absent in our proteome. Of the three others known pathogenicity factors of *Helicobacter cinaedi*—Cdt, AhpC, and HcaA—only the latter, *Helicobacter cinaedi* autotransporter protein (HcaA), showed a notable increase in the bacterial proteome, being 49% more abundant after infection of macrophage cells. In contrast, the other two factors, Cdt (cytolethal distending toxin) and AhpC (alkyl hydroperoxide reductase), were present in the proteome but at reduced levels following infection compared to bacteria grown under culture conditions (Figure 3B). Specifically, the levels of Cdt subunits A, B, and C decreased by 42, 70, and 72%, respectively, while the abundance of AhpC was reduced by 20%.

Alkyl hydroperoxide reductase (AhpC) is an important enzyme that protects against oxidative stress; however, its role in *H. cinaedi* appears to be strain-dependent. A previous study (Charoenlap et al., 2012) showed that an AhpC-deficient mutant of *H. cinaedi* exhibited greater resistance to hydrogen peroxide (H_2_O_2_) than the wild-type strain, likely due to compensatory catalase activity—an enzyme that decomposes H_2_O_2_ to water and oxygen. In our proteomic analysis of *H. cinaedi* strain BAA-847, grown both under culture conditions and following macrophage infection, catalase levels remained relatively constant (Figure 3B). We can hypothesise that the current catalase activity was sufficient for the survival of the bacterium under our conditions. It is also possible that in addition to catalase, some other bacterial factors are involved in the defense against oxidative stress, which are not yet known.

Cytolethal distending toxin (Cdt) induces cell cycle arrest and apoptosis in eukaryotic cells, with macrophages reportedly being an order of magnitude more susceptible than epithelial and endothelial cells (Jinadasa et al., 2011). While Cdt has been implicated in *H. cinaedi* intestinal invasion (Shen et al., 2009), its role in macrophage interaction remains unclear. In our study, the reduced abundance of Cdt in the bacterial proteome following macrophage infection suggests that this toxin may not be actively involved in the early stages of *H. cinaedi*-macrophage interaction. One possible explanation is that upon infection, the bacterium prioritizes intracellular survival over host cell apoptosis. It is also plausible that our observations capture an early infection stage, within the first 24 h, during which Cdt has not yet been upregulated or required for macrophage apoptosis. Further time-course studies are warranted to determine whether Cdt plays a delayed role in modulating macrophage viability.

### The main mechanisms involved in *Helicobacter cinaedi* survival during infection of macrophage cells

The 109 most prominently represented proteins in the *H. cinaedi* proteome following macrophage infection (Supplementary Table 2) were categorized into functional groups (Figure 3C). Notably, these groups often overlap; for example, proteins involved in membrane transport and gene regulation may both contribute to survival adaptations within macrophages. Thus, the classification presented in Figure 3C should be viewed as one possible scheme for organizing these proteins by function.

The largest subset of upregulated proteins post-infection is associated with membrane transport, metabolic adaptations for intracellular survival, and nucleic acid-related processes, including gene regulation, transcription, and DNA repair. Other prominent functional categories include proteins involved in cell wall biosynthesis, defense mechanisms, bacterial motility, and the operation of secretion systems (Figure 3C).

**Figure 4:**
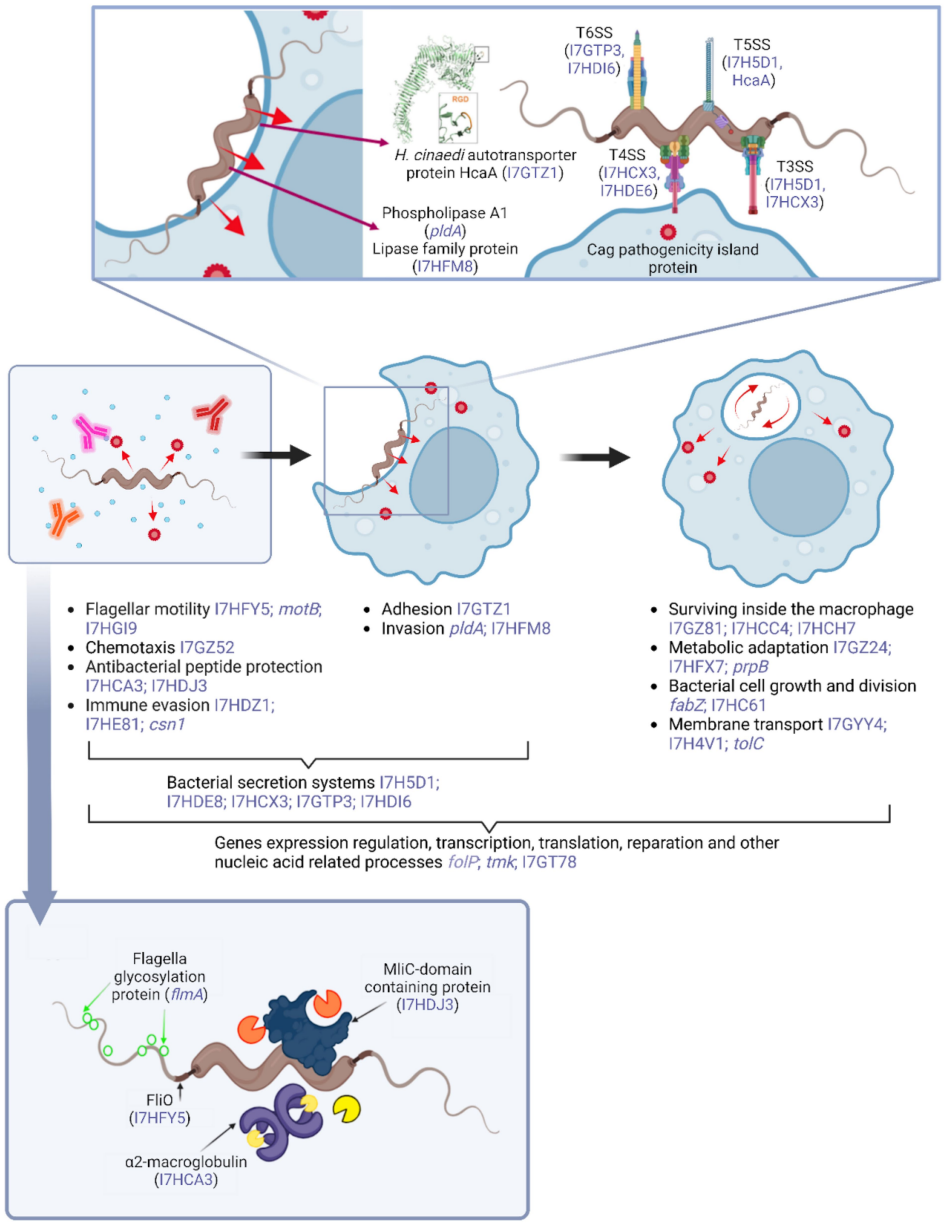
Schematic representation of the hypothetical processes involved in the interaction between *H. cinaedi* and macrophage cells within the host organism. The diagram highlights key stages of infection and bacterial adaptation, along with examples of potential pathogenicity factors identified in this study that show significantly increased representation following macrophage infection (indicated in blue font). For proteins lacking an assigned gene name, UniProt accession numbers are provided, also in blue font. Figure created with BioRender.com.

During the initial stages of *H. cinaedi*–macrophage interaction in the host, motility, chemotaxis, resistance to antimicrobial peptides, and phase variation are critical for immune evasion (Figure 4). These are followed by adhesion and invasion into macrophages, as described by Ribet and Cossart (2015). Bacterial secretion systems are likely involved throughout these stages. Once internalized either via bacterial invasion mechanisms of phagocytosis *H. cinaedi* activates diverse metabolic pathways to adapt to the hostile intracellular environment. In tandem, membrane transport systems facilitate the uptake of essential nutrients such as carbon, nitrogen, and trace metals (Ehtram et al., 2021; Prajapati et al., 2021). Concurrently, adaptation to intracellular conditions must support bacterial growth and division, making cell wall synthesis another essential process. All the aforementioned processes ultimately rely on the regulation of gene expression, transcription, translation, and various DNA repair and other processes related to nucleic acids. These processes are basic and universal for the functioning of a bacterial cell, as well as for its adaptation to new conditions—therefore, proteins of this functional group prevail among the most elevated after infection of macrophage cells.

Below we will discuss how 109 *H. cinaedi* proteins elevated after infection of macrophages may be involved in the mechanisms of *H. cinaedi* pathogenicity. The description of the features of each group of virulence factors and their functioning is made based on the *H. pylori* example, as the most studied of the *H. cinaedi* related microorganisms.

### Bacterial motility and chemotaxis

Motility is an important characteristic because it allows pathogens to move towards more favorable conditions and colonize host cells (Palma et al., 2022). In the case of *H. pylori*, it has long been known that flagellar motility plays an important role in the initial stage of host colonization: artificially engineered flagellineless mutants show a reduced ability to infect mice compared to wild-type bacteria (Ottemann and Lowenthal, 2002). Genes encoding proteins of the flagellar apparatus, such as *flaA, flaB*, *fliD*, *fliQ*, *flhB* are responsible for bacterial motility (Foynes et al., 1999; Kim et al., 1999; Josenhans et al., 1995).

Probably, flagellar motility as well as chemotaxis play a similar role in the case of *H. cinaedi*. A methyl-accepting chemotaxis protein (I7GZ52), responsible for chemotaxis, is present in the proteome after infection of macrophage cells. In addition, a number of proteins associated with flagellar formation are also present, including the named FliO protein (I7HFY5) (Figure 4). Another of the flagellar-associated proteins, flagellar glycosylation protein (I7HE93), contributes to evasion of the immune response through post-translational glycosylation of flagellin. Because flagellin is a highly immunogenic protein recognized by the host during infection, glycosylation promotes immune evasion and invasion (De Maayer and Cowan, 2016; Nothaft and Szymanski, 2010).

### Defense against antibacterial peptides and proteins

In order to kill pathogenic bacteria, macrophages can produce antimicrobial proteins and peptides such as cathelicidin (Boparai and Sharma, 2020; Rosenberger et al., 2004). These low molecular weight proteins are capable of disrupting the integrity of the microbial envelope by attaching to negatively charged membrane molecules, leading to cell death by osmotic shock (Sun et al., 2017), as well as disrupting bacterial biofilms, thereby reducing antibiotic resistance in bacteria (Pfalzgraff et al., 2018).

We detected an alpha-2-macroglobulin family N-terminal region protein in the *H. cinaedi* proteome after infection of macrophage cells (I7HCA3). According to the results of studies, bacterial alpha-2-macroglobulins belong to the “rudimentary immune system” and are able to mimic the initial stages of the innate immune response of eukaryotes, cleave antibacterial proteases of the host organism, and inhibit enzymes of competing bacteria and phages (Wong and Dessen, 2014). Therefore, the *H. cinaedi* protein we detected appears to have a protective function by inhibiting antibacterial proteases (Figure 4). Another factor detected, MliC domain-containing protein (I7HDJ3), is an inhibitor of c-type lysozyme and protects the bacterium from the effects of lysozymes that degrade cell wall peptidoglycans (Callewaert et al., 2008; Yum et al., 2009). Lysozymes are secreted by macrophages to kill bacteria within phagosomes, but in addition, during *in vivo* infection, lysozyme is secreted by epithelial cells to fight bacteria and activate macrophages (Ragland and Criss, 2017). Thus, in our experiment, the MliC domain-containing protein likely begins to function after invasion and should be considered as a component of adaptation to survival within macrophages. However, in a broader sense, during infection in vivo, this factor may exert protective functions even before the stage of adhesion and penetration of the bacterium into eukaryotic cells.

### Phase variability

One of the mechanisms for evading host immune response in pathogenic bacteria, including *H. pylori*, is phase variability, an adaptive process in which there are frequent and reversible phenotypic changes in the expression of surface antigens due to genetic changes at specific loci in their genome (Gauntlett et al., 2014; Rao et al., 2014). Phase variability may be provided by changes in methyltransferase activity within a restriction-modification system (R-M). Another known mechanism in *H. pylori* is phase variability by changing the amount and/or position of tryptophan in proteins at the level of the genetic code, with most exchanges occurring in membrane and cation-binding proteins (Rojas-Rengifo et al., 2019).

The proteins we detected in the proteome of *H. cinaedi* after infection of macrophage cells indicate that this species also utilizes phase variability to evade the immune system response. For example, two type III R-M proteins (I7HDZ1 and I7HE81) and anthranilate phosphoribosyltransferase (I7HCE8), a protein involved in tryptophan biosynthesis, are present in the proteome. Another protein, CRISPR-associated endonuclease Cas9 (I7GTK8), may also contribute to phase variation and adaptation by regulating the production of immunogenic proteins via an antisense RNA-based silencing mechanism (Louwen et al., 2014).

### Factors contributing to *Helicobacter cinaedi* adhesion

For successful colonization of the host, bacteria must overcome tissue barriers and adhere to the surface of epithelial cells. *H. pylori*, for instance, possesses several proteins that facilitate attachment to epithelial surfaces, including the blood group antigen-binding adhesin BabA, the outer membrane protein HopZ, and the sialic acid-binding adhesin SabA (Mahdavi et al., 2002; Marcus et al., 2018; Baj et al., 2020).

Recent studies by Sae Aoki’s group have extensively characterized the HcaA protein (I7GTZ1), which plays a critical role in the adhesion of *H. cinaedi* to eukaryotic cells (Figure 4) (Aoki et al., 2023). Notably, the abundance of HcaA significantly increases in the bacterial proteome following infection of macrophage cells, suggesting a key role in facilitating adhesion to macrophage surfaces. It is important to highlight that HcaA is a component of the Type V secretion system (T5SS) and functions as an autotransporter. Therefore, it is plausible that other autotransporters identified in the proteome may also contribute to the adhesion process.

### Factors contributing to *Helicobacter cinaedi* invasion

After *H. pylori* colonizes the host tissue surface, it employs strategies to penetrate deeper tissues or host cells, promoting immune evasion and long-term survival. Intracellular localization allows *H. pylori* to avoid recognition by the host immune system (Amieva et al., 2002). To facilitate invasion, *H. pylori* secretes the VacA toxin, which disrupts the epithelial barrier (Jeyamani et al., 2018; Baj et al., 2020). Additionally, *H. pylori* produces several types of phospholipases—phospholipase A1, A2, C, and D (Figure 4) that contribute to host cell membrane disruption through phospholipid hydrolysis (Berstad et al., 2002). These enzymes, commonly located in the outer membrane of Gram-negative bacteria, play a critical role in the invasion process (Istivan and Coloe, 2006).

Phospholipase A1 (I7HG31) and a lipase family protein (I7HFM8) are present in the proteome of *H. cinaedi* following infection of macrophage cells. Lipases are known to facilitate the destruction of host cell membranes during invasion of eukaryotic cells, aiding bacterial entry (Ruiz et al., 2007). Additionally, a TIR domain-containing protein (I7HGH6) may contribute to *H. cinaedi* adhesion and invasion, thereby promoting immune evasion. This bacterial protein is an analog of the TIR domain found in Toll-like receptors (TLRs) of immune cells. By competing for binding to adaptor proteins essential for TLR signaling, it interferes with downstream signaling and suppresses NF-κB activation, impairing the host immune response (Rana et al., 2013).

### Bacterial secretion systems

Bacterial secretion systems are specialized protein complexes that transport bacterial metabolites or effector molecules across the cell membrane (Naskar et al., 2021). In Gram-negative bacteria, six major secretion systems have been identified—Type I (T1SS) through Type VI (T6SS)—along with the Sec and Tat translocation pathways, which assist in the proper functioning of the Type II (T2SS) and Type V (T5SS) systems (Green and Mecsas, 2016). These systems differ in structural complexity and mode of action. While T1SS, T2SS, and T5SS primarily export proteins to the extracellular environment, T3SS, T4SS, and T6SS deliver effector proteins directly into host cells. During intracellular persistence, secretion systems may also facilitate the export of proteins involved in iron acquisition or the formation of pores in host cell membranes, supporting bacterial survival and proliferation (Maphosa et al., 2023).

In the *H. cinaedi* proteome following macrophage infection, components of multiple secretion systems were detected, including T3SS, T4SS, T5SS, and T6SS (I7H5D1, I7HDE8, I7HCX3, I7GTZ1, I7GTP3 and I7HDI6). It should be noted that some transport proteins identified in the proteome, such as the Sec-independent protein translocase TatB (I7H591), may also be functionally associated with bacterial secretion systems.

### Metabolic adaptation for *Helicobacter cinaedi* survival inside macrophage cells

Bacterial adaptation for survival within macrophages and other eukaryotic cells is a complex and multifaceted process. In addition to mounting defenses against host-derived bactericidal factors—such as reactive oxygen species (ROS), antimicrobial peptides, and acidic pH—bacteria must also overcome nutrient limitation by securing essential elements including carbon, nitrogen, and metal ions (Marshansky and Futai, 2008; Uribe-Querol and Rosales, 2017; Valenta et al., 2020). For instance, host defense mechanisms actively sequester divalent metal ions like Fe^2+^, which are critical for bacterial metabolism. Proteins such as lactoferrin and the NRAMP-1 transporter remove Fe^2+^ from the phagolysosomal compartment, thereby restricting microbial access to this essential nutrient (Cellier et al., 2007; Ward et al., 2011). To persist within such an environment, intracellular pathogens must develop mechanisms to circumvent host metal sequestration and maintain metabolic activity.

The topic of pathogen adaptation to the intracellular environment is extensive, and includes a variety of mechanisms such as retaining essential metal ions, fighting acidic conditions, and resisting reactive forms of nitrogen and oxygen (Dian et al., 2011; Nolan et al., 2002; Sidebotham et al., 2003; Benoit and Maier, 2016; Lewis et al., 2010).

*Helicobacter cinaedi*’s adaptation to iron deficiency may be facilitated by a putative heme iron utilization protein (I7GZQ5). The ability to extract iron from heme is a known survival strategy employed by Gram-negative pathogens under unfavorable conditions (Richard et al., 2019). Additionally, the enzymes 3,4-dihydroxy-2-butanone 4-phosphate synthase (I7GYU3) and GTP cyclohydrolase-2 (I7HF58) are upregulated in the proteome following infection of macrophage cells. These enzymes catalyze essential steps in the riboflavin biosynthesis pathway, which is critical for bacterial survival in iron-limited environments (Richter et al., 1997; Worst et al., 1998; Ren et al., 2005). A ferritin-like domain-containing protein (I7HCC4) is also elevated and belongs to a multifunctional family of ferritin-like compounds that can store iron, protect against iron-induced oxidative stress, serve as an iron reservoir under deficiency, and shield DNA from enzymatic or oxidative damage (Smith, 2004).

Superoxide dismutase (I7GZ20) and nitrite reductase (I7HCH7) likely function to protect *H. cinaedi* from oxidative stress during survival within the hostile environment of the macrophage lysosome (Poock et al., 2002; Clarke et al., 2008; Broxton and Culotta, 2016). Additionally, L-asparaginase (I7GSX0) may contribute to the modulation of pH, potentially aiding in bacterial adaptation to the acidic conditions of the phagolysosome (Belén et al., 2022).

The C4-dicarboxylate-binding periplasmic protein (I7GZ24) is involved in the transport of C4-dicarboxylates (C4-DCs). These molecules, such as succinate, are produced by host cells—including macrophages—and can be utilized by invading bacteria as alternative nutrient sources. This uptake supports bacterial survival under nutrient-limited conditions. Notably, the internalization of succinate has been shown to induce the expression of virulence factors, including components of the pathogenicity islet, which are critical for intracellular survival (Schubert and Unden, 2022). Thus, this protein may not only enhance the metabolic adaptability of *H. cinaedi* under stress conditions but also potentiate its pathogenic capabilities within host cells.

For nitrogen acquisition, *H. cinaedi* appears to utilize a clan AA aspartic protease (I7HG93), an enzyme capable of cleaving host eukaryotic proteins to release digestible nitrogen sources (Theron and Divol, 2014). In addition, enzymes involved in the initial steps of branched-chain amino acid (BCAA) biosynthesis—threonine dehydratase (I7H637) and ketol-acid reductoisomerase (I7H6E2)—are upregulated in the *H. cinaedi* proteome following macrophage infection. BCAAs such as isoleucine, leucine, and valine are essential for protein synthesis, signal transduction, and adaptive responses to amino acid starvation, a condition that can trigger virulence gene expression in certain pathogens (Kagan and Dorozhko, 1973; Chen et al., 2018; Kaiser and Heinrichs, 2018). Consequently, the synthesis of these amino acids may contribute to *H. cinaedi*’s ability to proliferate during infection and evade host immune responses.

Many of these adaptive mechanisms are likely mediated by specialized transport systems, including ABC transporters (I7GTC7, I7H4Q9, I7H4Q4 and I7GYM4) and outer membrane transport proteins (I7H4K4 and I7GZ13), which facilitate the uptake of essential nutrients and export of toxic compounds (Nikaido and Takatsuka, 2009; Zgurskaya et al., 2011; Noster et al., 2019; Akhtar and Turner, 2022).

### Bacterial division

Bacterial division is an integral part of the normal bacterial cell cycle (Harry et al., 2006). However, during infection, an increase in the bacterial population provides more material for natural selection, as phase variations can occur among a greater number of cells, enhancing the survival of the pathogen. Additionally, under unfavorable conditions, bacterial cells may become deficient in nutrients essential for growth and the normal division cycle.

In this context, while specific factors associated with the growth and division of *H. cinaedi* may not be considered pathogenicity factors per se, they undoubtedly contribute to the overall process of bacterial infection. This could explain the observed increase in the abundance of proteins involved in bacterial division and cell wall formation in the proteome following infection of macrophage cells.

### Factors that may be related to foam cell formation and *Helicobacter cinaedi*-associated atherosclerosis

Our proteomic data allow us to hypothesize what mechanisms and specific factors of *H. cinaedi* may induce the degeneration of macrophage cells into foam cells and, as a consequence, participate in the development of *H. cinaedi*-associated atherosclerosis.

The main cause of the foam cells formation is the disruption of natural lipoprotein metabolism within macrophages (Gui et al., 2022). It may occur due to several factors, such as enzyme malfunction or an imbalance between the capture and pumping of cholesterol out of cells (Chistiakov et al., 2017). Intracellular pathogens can also influence host cell metabolism, for example through the functioning of secretion systems (Maphosa et al., 2023).

In the *H. cinaedi* proteome, several types of secretion systems have been identified including T4SS as well as Cag pathogenicity island-associated protein (I7HDE8) (Figure 4). In *H. pylori*, the CagA protein translocates into host cells via T4SS and disrupts host cellular processes by interacting with multiple signaling molecules (Hatakeyama, 2017; Cover et al., 2020). It is likely that *H. cinaedi* utilizes the T4SS to deliver the Cag protein into macrophage cells, potentially disrupting intracellular metabolic processes and promoting bacterial survival. Although the specific functions of *H. cinaedi* Cag protein in macrophages remain unclear, CagA from *H. pylori* has been shown to induce apoptosis in these immune cells (Menaker et al., 2004). Based on this, we hypothesize that the *H. cinaedi* Cag protein may contribute to the formation of foam cells—an effect clearly observed following infection—suggesting a possible role in modulating host lipid metabolism and immune responses.

Another mechanism of interest in exploring the role of *H. cinaedi* in foam cell formation is the methylcitrate cycle, which is responsible for the oxidation of propionate to pyruvate. Enzymes such as 2-methylcitrate dehydratase (I7HFX7) and 2-methylisocitrate lyase (I7GZG5) are key components of this pathway. The methylcitrate cycle has been implicated in the survival of intracellular pathogens by enabling the utilization of cholesterol as a primary carbon source within macrophages (Aoki and Tabuchi, 1981; Brock et al., 2001, 2002; Dolan et al., 2018). While further investigation is necessary, it is plausible to hypothesize that the accumulation of low-density lipoproteins (LDL) and cholesterol in macrophages may be beneficial for the intracellular persistence of *H. cinaedi*, as these lipids could serve as accessible nutrients. In this context, *H. cinaedi* may possess mechanisms that promote or exploit cholesterol accumulation within host macrophages, potentially contributing to foam cell formation.

Unfortunately, to date, most of the factors identified in this *H. cinaedi* proteome have been poorly characterized and additional studies are required to better understand foam cell-inducing mechanisms.

### Limitations and future study directions

Our study has some limitations. The main limitation of our study is that it is not possible to validate the results obtained by mass spectrometric methods by any alternative method. Since no antibodies are available for the *H. cinaedi* proteins we identified, it is not possible to validate them by Western blot analysis. Also, due to the different intracellular processes between transcription and translation, we do not consider it relevant to correlate our results on protein representation with any transcriptome analyses. We find it more interesting to test the potential pathogenicity of the identified factors through gene knockout (mutagenesis studies), conditional expression studies and infection model studies, which we are planning to perform in our future research.

Another limitation of the present results is the use of a certain (24 h) incubation time of *H. cinaedi* with macrophages and cultivation (7 days) of the bacterial culture before proteome isolation. Using a different period of incubation or bacterial culture buildup could lead to different results, since changes in the *H. cinaedi* proteomic profile after infection of macrophages are certainly time-dependent.

## Conclusion

In this study, we provide a comprehensive analysis of the *H. cinaedi* proteome, which, to the best of our knowledge, has not been previously described in the literature. A total of 1,575 proteins, representing approximately 68% of the 2,322 protein-coding genes of *H. cinaedi* strain BAA-847, were identified across seven samples. Despite its medical relevance, *H. cinaedi* remains poorly understood, particularly regarding the protein factors responsible for inducing the foam cell phenotype. In addition to foam cell formation, several other mechanisms of interaction between the bacterium and macrophage cells remain of significant interest, including immune evasion, invasion, and survival strategies within macrophages. By analyzing changes in the bacterial protein profile, we aim to shed light on these mechanisms.

The data presented here may also contribute to further understanding of *H. cinaedi* pathogenicity, particularly in terms of its interactions with intestinal cells when the bacterium translocates from the gastrointestinal tract into the bloodstream (Aoki et al., 2023). Additionally, we provide detailed data on the growth dynamics of *H. cinaedi*. To our knowledge, no previous studies have published a CFU counting methodology or the growth dynamics of this bacterium. We believe that this information will be valuable for future experimental work involving *H. cinaedi*.

## Data Availability

The mass spectrometry proteomics data have been deposited to the ProteomeXchange Consortium via the PRIDE partner repository with the dataset identifier 10.6019/PXD064480.

## Footnotes

^1^https://www.who.int/health-topics/cardiovascular-diseases

^2^http://www.uniprot.org/

## Supplementary material

SUPPLEMENTARY FIGURE 1 **(A,B)** Determination of the minimum inhibitory concentration (MIC) of gentamicin. Gentamicin concentrations in each vial are signed in μg/mL in the photo. **(C)** Bacterial CFU counting on 4th day. **(D,E)** Bacterial CFU counting on 7th day. Scale bar = 1 mm.

SUPPLEMENTARY TABLE 1 Summary table of proteins and tryptic peptides identified in culture conditions of *H. cinaedi* and after infection of macrophage cells.

SUPPLEMENTARY TABLE 2 A brief characterization of proteins with increased representation in *H. cinaedi* bacterium following infection of macrophage cells.
