# Supplementary figures and images for: Changes in potential pathogenicity-associated proteins of Helicobacter cinaedi upon infection of macrophage cells
Source: Front Microbiol. 2025 Aug 25;16:1640829. doi: 10.3389/fmicb.2025.1640829 (PMC12415068; doi:10.3389/fmicb.2025.1640829)

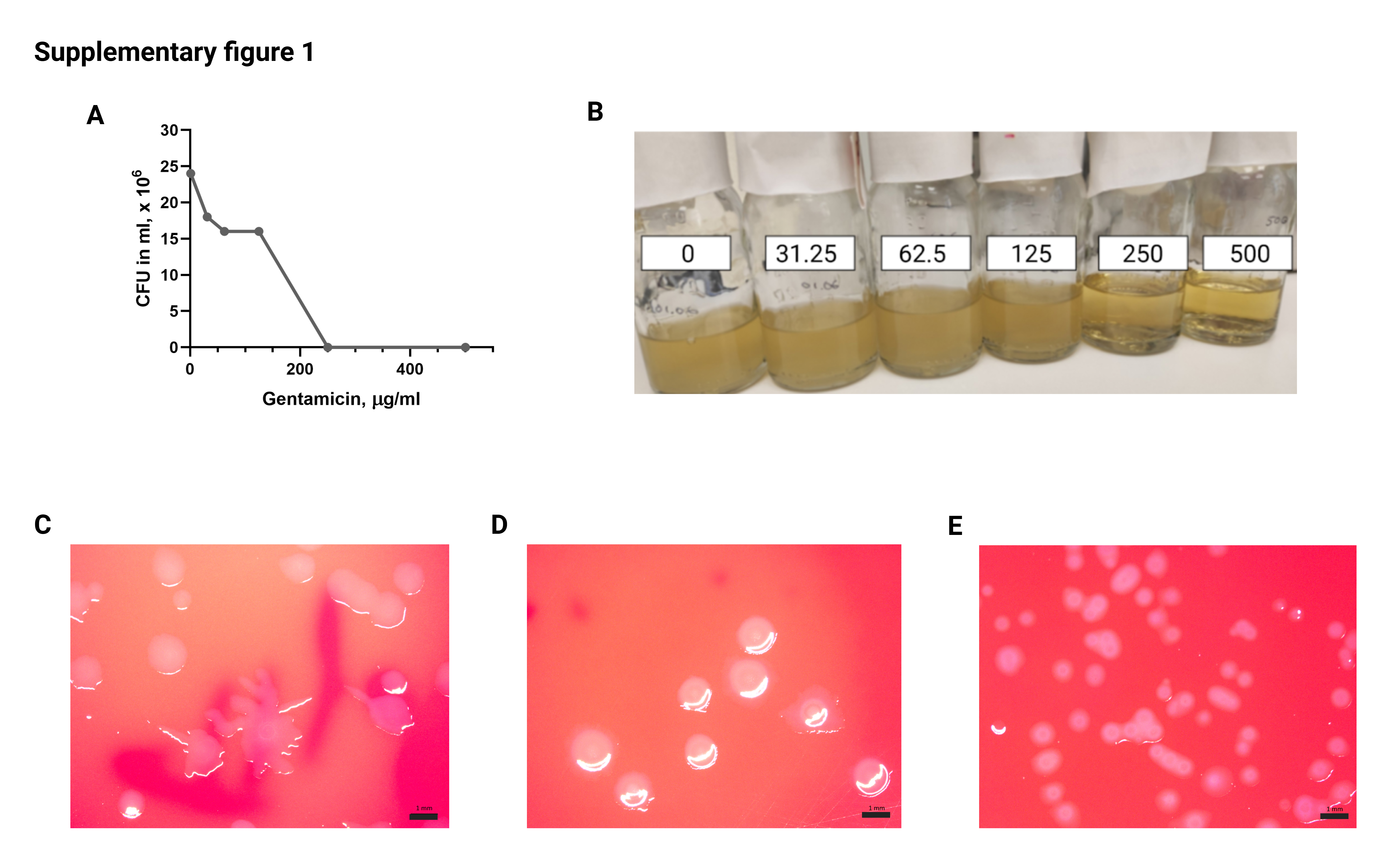

Supplement: SUPPLEMENTARY FIGURE 1 — (A,B) Determination of the minimum inhibitory concentration (MIC) of gentamicin. Gentamicin concentrations in each vial are signed in μg/mL in the photo. (C) Bacterial CFU counting on 4th day. (D,E) Bacterial CFU counting on 7th day. Scale bar = 1 mm. [file Image_1.jpeg]
